# Attaining Toughness and Reduced Electrical Percolation Thresholds in Bio-Based PA410 by Combined Addition of Bio-Based Thermoplastic Elastomers and CNTs

**DOI:** 10.3390/polym13193420

**Published:** 2021-10-05

**Authors:** Itziar Otaegi, Nora Aranburu, Gonzalo Guerrica-Echevarría

**Affiliations:** Department of Polymers and Advanced Materials—Physics, Chemistry and Technology & POLYMAT, Faculty of Chemistry, University of the Basque Country UPV/EHU, Paseo Manuel de Lardizabal 3, 20018 Donostia-San Sebastián, Spain; itziar.otaegi@ehu.eus (I.O.); nora.aramburu@ehu.eus (N.A.)

**Keywords:** polyamide, thermoplastic elastomer, biopolymer, blend, nanocomposite, carbon nanotube, toughness, electrical conductivity

## Abstract

Multi-walled carbon nanotubes (CNTs) were added to provide electrical conductivity to bio-based polymer blends with improved toughness (based on commercially available Pebax thermoplastic elastomers and bio-based polyamide 4,10). A preliminary study including three different Pebax grades was carried out to select the grade and the composition that would best improve the impact properties of PA410. Thus, tough multiphasic PA/Pebax/CNT nanocomposites (NCs) with enhanced electrical conductivity were obtained. The CNTs were added either: (1) in the form of pristine nanotubes or (2) in the form of a PA6-based masterbatch. Hence, PA410/Pebax/CNT ternary NCs and PA410/PA6/Pebax/CNT quaternary NCs were obtained, respectively, up to a CNT content of 1 wt%. The ternary and quaternary NCs both showed similar mechanical and electrical properties. The electrical percolation threshold decreased with respect to previously studied corresponding NCs without Pebax, i.e., PA410/CNT and PA410/PA6/CNT, due to the partial volume exclusion effect of Pebax over the CNTs that were dispersed mainly in the PA matrix; materials with percolation concentrations as low as 0.38 wt% were obtained. With respect to mechanical properties, contrary to the NCs without Pebax, all the PA/Pebax/CNT NCs showed a ductile behavior and impact strength values that were from three to five-fold higher than that of the pure PA410.

## 1. Introduction

Polyamides are one of the most widely used families of engineering polymers due to their good mechanical and thermal properties; their applications include the electrical, electronics and automobile industries [1,2,3]. Polyamides include a broad range of materials depending on the monomers used for their production. In many cases, commercial polyamides are obtained by condensation reactions between diamines and dicarboxylic acids, and a significant effort has been made to obtain these monomers from renewable resources. Dicarboxylic acids, for instance, can be obtained from plant oils, thus enabling the production of bio-polyamides [4]. In this sense, castor oil is one of the most interesting vegetable oils for the mass production of commercial bio-polyamides [5,6,7,8]. The bio-PA410 used in this study is produced from sebacic acid derived from castor oil, thus, achieving a polyamide with a renewable carbon content of about 70% and properties comparable to its nearest competitors, i.e., PA6 and PA66.

Similar to traditional polyamides, bio-polyamides also have outstanding mechanical properties [9], but the impact strength is still their main weakness. One of the best-known strategies to improve the toughness of polymeric materials with low impact strength—either because they are brittle or because they are notch sensitive—is blending them with elastomeric materials [10,11,12,13]. There is a wide variety of commercially available elastomers and the choice, obviously, depends on the nature of the matrix, since a minimum level of compatibility between the matrix polymer and the dispersed phase is needed; however, thermoplastic elastomers have gained significance in recent years [14,15,16,17,18]. The capability of thermoplastic elastomers to modify the matrix properties is usually more limited as compared with the traditional cross-linked elastomers [14]. Nevertheless, they present important advantages, for instance, the recyclability of elastomer scraps [14,15,16] or the processing ease and rapidity [15,16], with the subsequent savings in energy costs [15,16]. Therefore, they can be processed along with the matrix in a single step using the traditional techniques employed in the processing of thermoplastic polymers, such as extrusion and injection molding [14], which is a prevailing advantage in many cases.

To date, the elastomers used to improve the toughness of brittle polymers have generally been petrochemical, and very few bio-based or biodegradable elastomers have been studied. However, the study of bio-based elastomers has proven to be very interesting, considering that impact properties can be enhanced without sacrificing the renewable nature of the material. In this sense, the partially bio-based polyether-block-amide copolymers (Pebax) series is one of the most studied groups of bio-based thermoplastic elastomers [19,20,21,22,23], as they provide a wide range of flexibility, while maintaining high strength, toughness, and good processability [24].

Furthermore, polymer nanocomposites (NCs) based on polyamide and carbon nanotubes (CNTs) have attracted much interest in recent decades [25,26,27,28]. The most relevant contribution of CNTs in polymeric matrices is that they provide electrical conductivity to these inherently insulating materials [29]. The electrical conductivity is attained when the so-called electrical percolation threshold (p_c_) is achieved, i.e., when a three-dimensional network of interconnected nanotubes is formed in the polymeric matrix. Moreover, it is widely known that CNTs can improve the mechanical properties of polymeric materials. For this reason, the combination of thermoplastic elastomers and CNTs is of particular interest. The simultaneous addition of an elastomer and CNTs in a polymeric system can bring toughness and electrical conductivity, respectively; at the same time, the combination is very interesting from an engineering point of view. Moreover, the addition of an immiscible phase in a polymeric matrix can reduce the electrical percolation concentration due to a volume exclusion effect, thus, reducing the amount of CNTs needed to attain electrical conductivity. Gonzalez et al. [30], for instance, observed that in a PA6/CNT system the percolation threshold decreased from 4–5% to 3.5% after the addition of 30% of an immiscible elastomeric phase as a result of the volume exclusion effect. They obtained highly conductive, super-tough and very ductile materials with a modulus similar to that of pure PA6. In addition to the decrease in percolation threshold, the volume exclusion effect resulting from the presence of an immiscible phase can also cause increases in the values of electrical conductivity. Dasari et al. [31] observed that the electrical conductivity values of a PA6/CNT system increased after elastomer addition. What is more, they obtained super-tough and conductive materials by adding both 20% elastomer and 5% CNT. Some interesting reviews dealing with bio-based polymeric NCs have been recently published [32,33,34].

In this study, once the Pebax grade and composition that would best improve the properties of the pure PA410 was established, and in order to combine the benefits of adding Pebax and the advantages provided by CNTs, multiphasic PA/Pebax/CNT NCs were prepared. Two alternative processing routes were employed in preparing the multiphasic NCs. First, CNTs were added in pristine form, thus, obtaining PA410/Pebax/CNT NCs. Then, they were added in the form of a PA6/CNT masterbatch, which has previously been proven an efficient procedure to add CNTs to PA410 [1], thus, obtaining PA410/PA6/Pebax/CNT NCs. As a result, the aim of this study is to improve the mechanical and electrical properties of the pure PA410 in order to expand its fields of application by providing the material with certain properties, i.e., toughness and electrical conductivity, while maintaining its renewable attributes.

## 2. Materials and Methods

### 2.1. Materials

The PA410 used in this work was EcoPaXX^®^ Q150-D, kindly provided by DSM (Genk, Belgium).

In this study, we used three grades of commercially available and partially bio-based Pebax elastomers (Pebax^®^ Rnew^®^ 25R53 SP 01, 40R53 SP 01, and 63R53 SP 01). They were provided by Arkema (Colombes, France) and consist of flexible polytetramethylene oxide (PTMO) and rigid polyamide 11 (PA11) constituents [20], the latter being derived from renewable castor oil [21]. The difference between the three grades lies in the PTMO to PA11 ratio. The renewable amino-11 content is 17–21% in the first (named Pebax1), 44–48% in the second (Pebax2), and 77–81% in the third (Pebax3).

Two preliminary studies were performed to select the optimum grade and composition of the PA410/Pebax binary system, respectively. The results of these two studies are compiled in the Supplementary Information. With respect to the optimum Pebax grade, the selection was based on mechanical criteria. Table S1 shows the Young’s modulus, yield strength, strain at break, and impact strength values of all the compositions studied. Similar to other studies [11,35,36,37], by increasing elastomer content, the values of the low-strain mechanical properties (i.e., Young’s modulus and yield strength) decreased, whereas the values of the high-strain properties (i.e., ductility and toughness) increased. Impact strength significantly increased, with values 5 times higher with respect to that of pure PA410 for a 30% elastomer content. Among the grades studied, Pebax3 showed the lowest increases in impact strength, probably due to its higher rigid polyamide segment content. Small differences were observed between Pebax1 and Pebax2, so the latter was selected for its higher renewable bio-based content with respect to the former.

In the second preliminary study, the PA410-rich composition range of the PA410/Pebax2 system was studied in depth. Figures S1 and S2 show, respectively, the tanδ vs. temperature scans obtained by DMTA and representative micrographs obtained by SEM of all the compositions studied. Tables S2 and S3 show, respectively, the calorimetric parameters obtained by DSC and the Young’s modulus, yield stress, strain at break, and impact strength of all the compositions studied.

The main conclusions from this study were: (1) The PA410/Pebax2 system shows full immiscibility with some level of interfacial interaction between both components, probably related to the presence of polyamide groups in the matrix and the elastomer. (2) The crystalline structure of PA410 slightly changed on the blends, showing slightly lower *T_m_*-s and higher melting enthalpies, indicating a nucleating effect of the dispersed particles of Pebax2, as expected in other thermoplastic/elastomer [38,39,40] and PA/elastomer [41,42,43,44] blends. (3) The morphology corresponds to a biphasic blend and confirms that the two components are immiscible. A typical sea-island morphology up to the 70/30 composition and a close-to phase inversion morphology for higher Pebax2 contents were observed. Dispersed particle size was extremely small, much lower than 1 µm, showing slight increases in the 75/25 and 70/30 compositions and more significant increases at higher Pebax2 contents. (4) Confirming the mechanical results of the previous preliminary study, the value of the Young’s modulus and yield strength linearly decreased as the Pebax2 content increased, and strain at break maintained or even increased, depending on composition. More interestingly, the impact strength increased up to the 70/30 composition (5 times higher than that of pure PA410), but eventually decreased at higher Pebax2 contents.

As a result of both preliminary studies, PA410/Pebax2 70/30 composition was selected as the base composition for the preparation of the CNT-filled nanocomposites.

The pristine carbon nanotubes used were Nanocyl NC7000^TM^ (Sambreville, Belgium). Additionally, a PA6-based MWCNT masterbatch, also manufactured by Nanocyl (Sambreville, Belgium) and commercialized as Plasticyl^TM^ PA 1503, containing 15 wt% of the aforementioned NC7000^TM^ CNTs was also used to obtain the PA410-based NCs.

### 2.2. Composite Processing

**Scheme 1:**
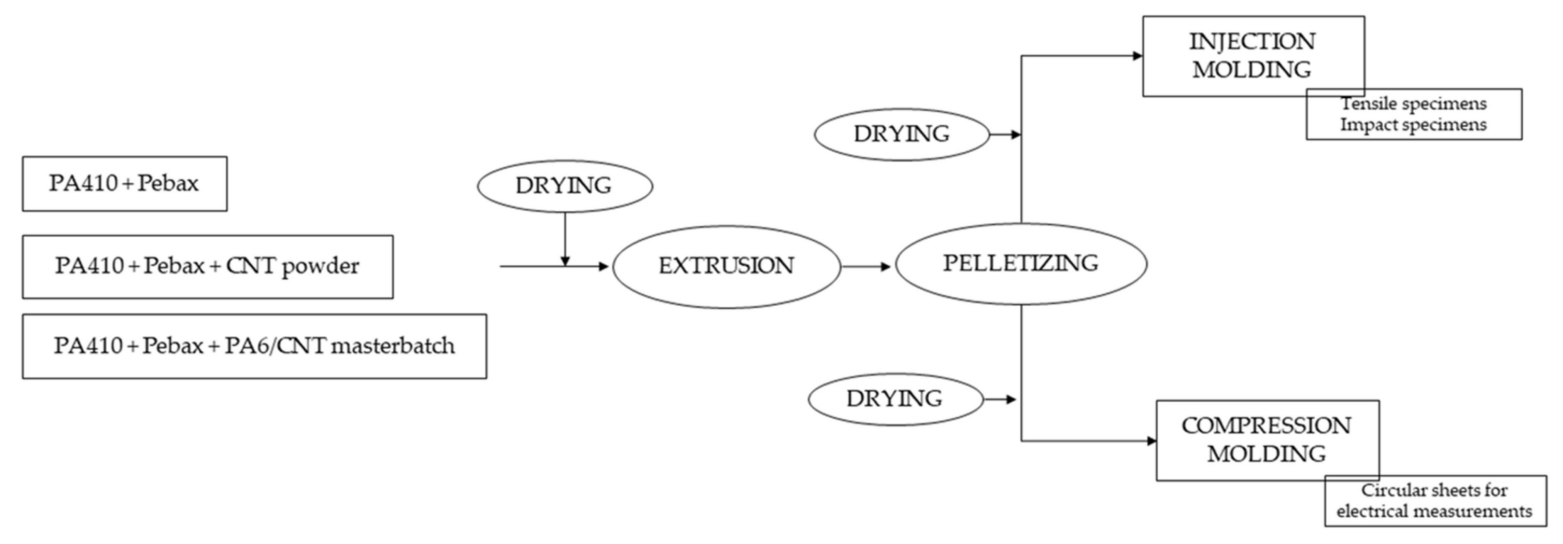
Schematic representation of the preparation of the materials.

Scheme 1 summarizes the processing steps carried out in the preparation of the materials. In order to prevent moisture-induced degradation reactions during processing, the PA410 and the Plasticyl^TM^ PA 1503 masterbatch were dried in a dry air dehumidifier (Wittmann Drymax, Kottingbrunn, Austria) for 48–72 h at 80 °C. The Pebax elastomers were dried in an air-circulation oven at 60 °C for 24 h. Unfilled CNT-free PA410/Pebax blends were obtained by melt mixing at 260 °C using a Collin ZK 25T SCD 15 Teach-Line corotating twin-screw extruder-kneader with a screw rotation speed of 200 rpm (Maitenbeth, Bavaria, Germany). The diameter and length-to-diameter ratio of the screws were 25 mm and 18, respectively. The CNT-filled PA410/Pebax/CNT ternary NCs were prepared by direct melt mixing of the pristine CNTs and the quaternary PA410/PA6/Pebax/CNT NCs were prepared using the masterbatch, which were also obtained under the same conditions using a Collin ZK25 corotating twin screw extruder-kneader (Maitenbeth, Bavaria, Germany). The diameter and length-to-diameter ratio of the screws were, in this case, 25 mm and 30, respectively. Table 1 shows the wt% content of each component for all the compositions studied.

Extruded materials were pelletized after cooling in a water bath. After drying again, they were injection molded in a Battenfeld PLUS 350/75 reciprocating screw injection molding machine (Kottingbrunn, Austria) with a press closing force of 350 kN. Tensile (ASTM D-638, type IV, thickness 2 mm) and impact (ASTM D-256, thickness 3.2 mm) specimens were obtained. The diameter of the screw in the plasticizing unit was 25 mm and the L/D ratio was 14. The melt and mold temperatures were, respectively, 260 °C and 30 °C. The injection speed, and pressure-holding and cooling times were, respectively, 42 cm^3^/s, 3 s, and 15 s. Postprocessing humidity absorption was avoided by keeping the specimens in a desiccator. 

Electrical conductivity measurements were performed using standard circular sheets (diameter and thickness, 70 mm and 1 mm, respectively). These sheets were obtained by hot pressing using a Collin P200E hydraulic press (Maitenbeth, Bavaria, Germany). Processing temperature and pressure were set at 260 °C and 130 bar, respectively. The molding process was carried out following this procedure: (1) preheating or plasticizing (closure without pressure, 2 min); (2) compression (closure under pressure, 3 min); (3) cooling under pressure (6 min). Therefore, the only property of the materials in the study that was not measured using injection molded tensile specimens was the conductivity. Although injection molding and hot-pressing techniques can be expected to produce different CNT dispersion levels, the methodology is still valid when the objective of the measurements is to compare the electrical conductivity of the materials obtained using different CNTs and preparation methods.

### 2.3. Phase Structure

DMA was used to study the phase behavior. A TA Q800 viscoelastometer (New Castle, DE, USA) that provided the loss tangent (tanδ) against temperature was employed. The single cantilever bending mode was selected and constant heating rate and frequency were set at, respectively, 4 °C/min and 1 Hz. The studied temperature interval ranged from −100 °C to 150 °C. Thermal properties related to melting and crystallization were studied by DSC using a Perkin-Elmer DSC-7 calorimeter (Waltham, MA, USA) calibrated using an indium standard as a reference. First heating scan was performed from 30 °C to 300 °C at 20 °C/min, and then cooled at the same rate. The melting and crystallization temperatures (*T_m_*, *T_c_*) were determined, respectively, from the maxima of the corresponding peaks during the heating and cooling scans, and the melting and crystallization enthalpies were determined from the areas under each of these peaks. The crystallinity of PA410 was calculated from the melting and cold crystallization enthalpies, considering an enthalpy of a 100% crystalline ($∆H_{f}^{\infty }$) PA410 of 269 J/g [45].

### 2.4. Morphology

Cryogenically fractured surfaces of tensile specimens were observed by scanning electron microscopy (SEM) after gold coating. A Hitachi S-2700 electron microscope (Fukuoka, Japan) was used at an accelerating voltage of 15 kV.

The nanostructure was analyzed by transmission electron microscopy (TEM). The samples were obtained from injection-molded specimens which were ultrathin sectioned at ~100 nm using a Leica EMFC 6 ultramicrotome equipped with a diamond knife. The micrographs were taken in a Tecnai G2 20 twin apparatus (FEI, Waltham, MA, USA), operating at an accelerating voltage of 200 kV.

### 2.5. Mechanical Properties

An Instron 5569 tensile tester (Norwood, MA, USA) was used for the tensile tests. Young’s modulus was measured at a crosshead speed of 1 mm/min using an extensometer. The crosshead speed for the determination of the yield stress and ductility, measured as the break strain (ε_b_), was 10 mm/min. A minimum of five tensile specimens were tested for each reported value.

Izod impact tests were carried out on notched specimens using a Ceast 6548/000 pendulum (Norwood, MA, USA). The notches (depth 2.54 mm and radius 0.25 mm) were machined after injection molding. A minimum of eight impact specimens were tested for each reported value.

### 2.6. Electrical Properties

Electrical conductivity values were obtained from the measured volume resistances, according to the ASTM D4496-87 standard. A Keithley 6487 picoammeter (Cleveland, OH, USA) and a Keithley 8009 Resistivity Test Fixture at 1 V were used for the measurement. Three measurements were performed for each reported value.

## 3. Results and Discussion

As explained in the experimental section, the CNTs were available both as pristine CNTs (powder) and as a PA6-based masterbatch, and therefore PA410/Pebax/CNT ternary NCs and PA410/PA6/Pebax/CNT quaternary NCs were prepared, respectively (Table 1).

### 3.1. Phase Structure

**Figure 1:**
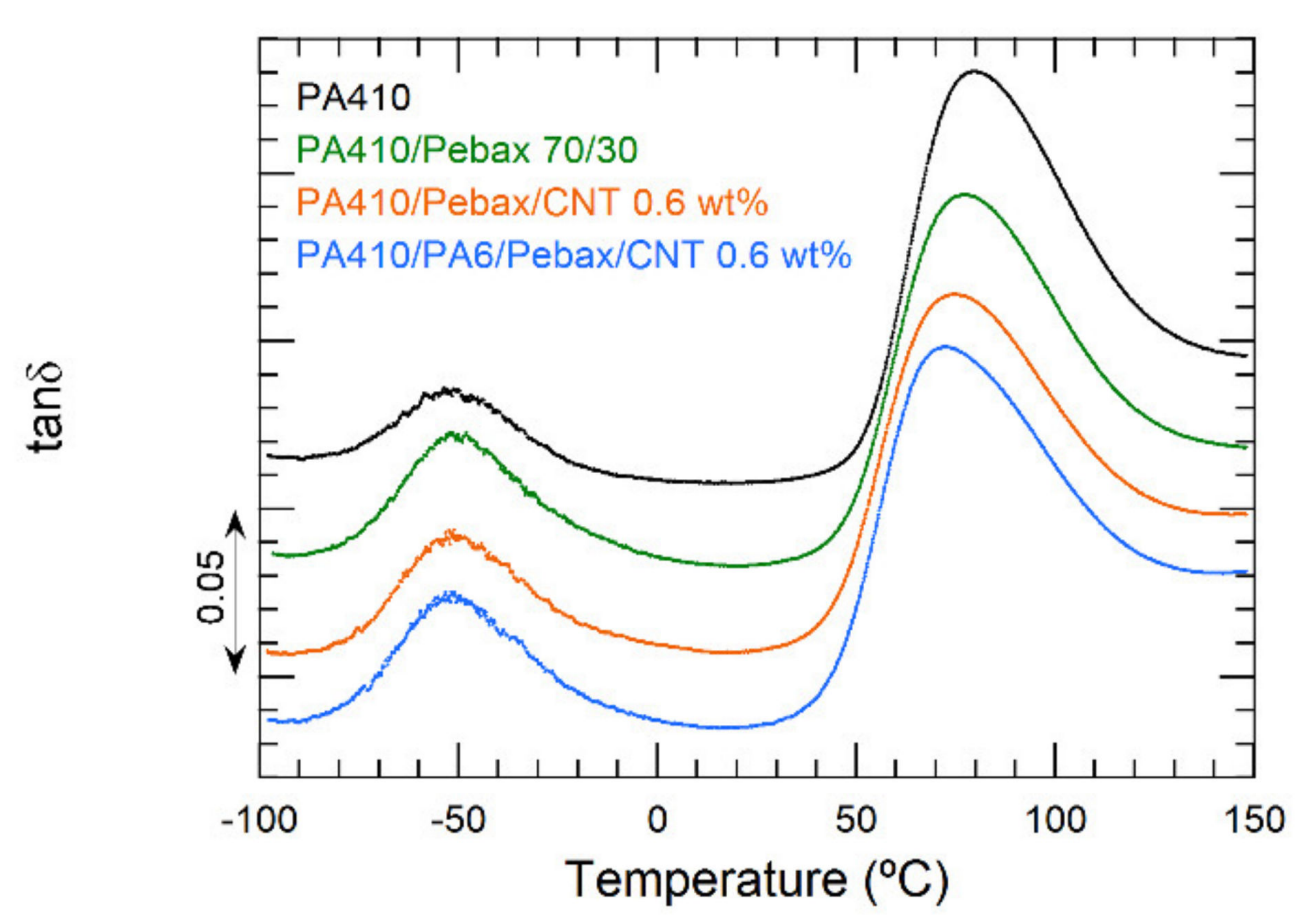
DMTA curves of pure PA410, PA410/Pebax 70/30 unfilled blend and, as an example, PA410/Pebax/CNT and PA410/PA6/Pebax/CNT NCs with a 0.6 wt% CNT content. Curves have been shifted in the vertical axis.

Figure 1 and Table 2 show the results obtained by DMTA. On the one hand, Figure 1 shows the tanδ vs. temperature curves of pure PA410, PA410/Pebax 70/30 unfilled blend and, as an example, PA410/Pebax/CNT and PA410/PA6/Pebax/CNT NCs containing 0.6 wt% CNTs. On the other hand, Table 2 shows the high temperature and low temperature peak positions of the tanδ vs. temperature thermograms for the PA410/Pebax/CNT and the PA410/PA6/Pebax/CNT systems. Those peaks correspond, respectively, to the T_g_ of the PA410 and to the superposition of the T_g_ of the Pebax2 and the β transition of the PA410. As can be observed, in the PA410/Pebax/CNT ternary system, the position of the high temperature peak hardly changes with the CNT content, and the decrease observed in T_g_ is very slight with respect to the pure PA410. Previous studies [1,46] have shown that the addition of CNTs causes slight increases in the T_g_; on the contrary, the addition of Pebax causes slight decreases in the T_g_ (see Figure 1 and Figure S1). Therefore, the small decrease observed in the ternary system is, apparently, the result of the overlapping of both effects. With respect to the low temperature peak, the tendency observed is similar to that of the unfilled PA410/Pebax system and corresponds to the simple superposition of the β transition of PA410 and the T_g_ of Pebax.

Regarding the quaternary PA410/PA6/Pebax/CNT system, and with respect to the ternary PA410/Pebax/CNT system without PA6, the decrease observed in the high temperature peak position is more significant in the former. Undoubtedly, this decrease is caused by the increasing PA6 content that increases with the CNT content and causes considerable decreases in the T_g_ of PA410/PA6 blends as a result of the miscibility of both polyamides, as evidenced in previous studies [46,47]. If the comparison is made with respect to the PA410/PA6/CNT system without Pebax [46], the decrease is slightly more evident in the quaternary PA410/PA6/Pebax/CNT system as a result of the contribution of the presence of Pebax, which causes slight decreases in the T_g_ (see Figure 1 and Figure S1).

Thus, DMTA results indicate that a simple addition of the effects of each component occurs both in the ternary and in the quaternary systems. There is no evidence, for instance, that Pebax has any influence over the miscibility of PA410 and PA6, or that PA6 affects in any way the immiscibility between PA410 and Pebax.

**Figure 2:**
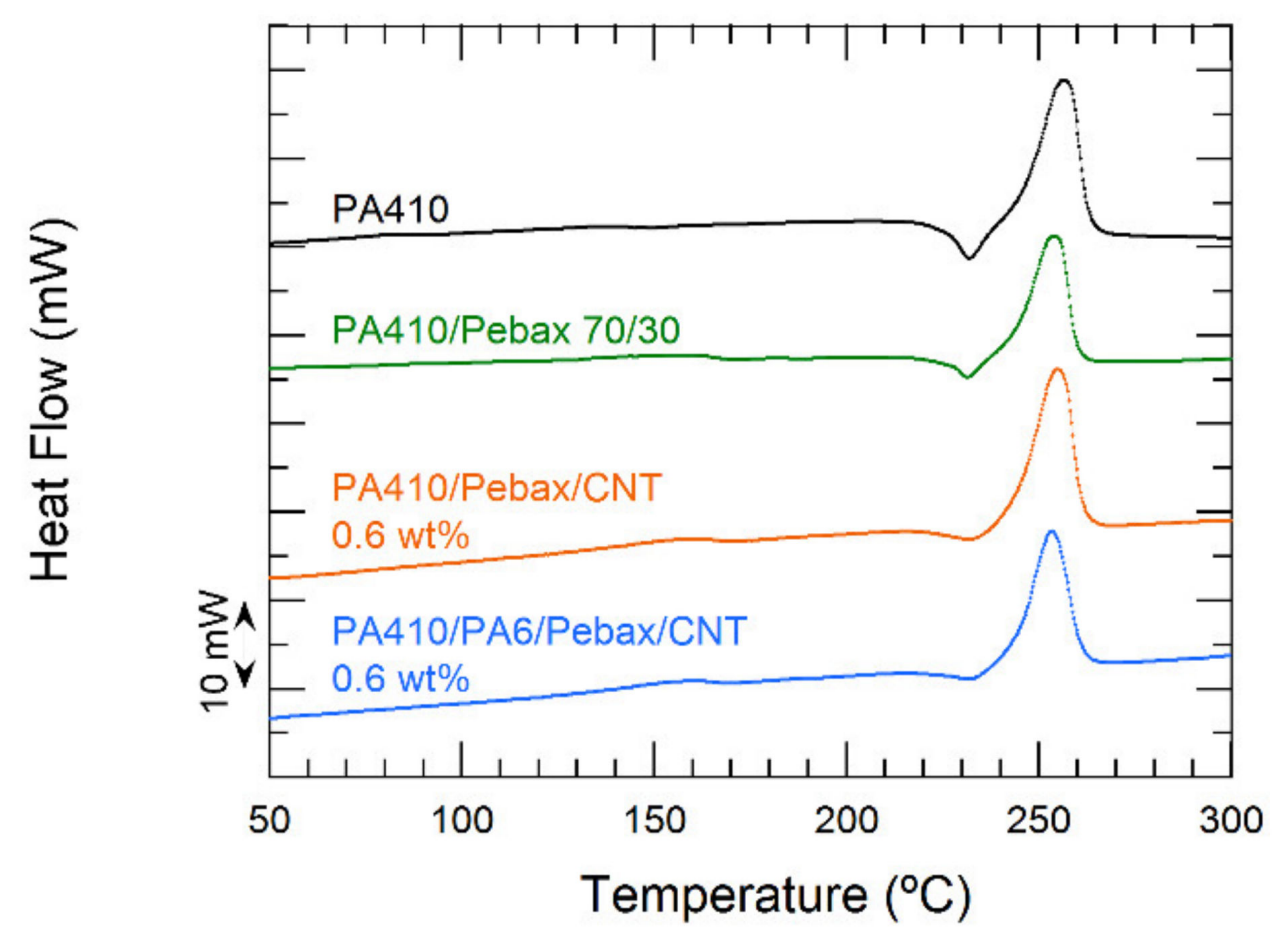
DSC curves of pure PA410, PA410/Pebax 70/30 unfilled blend and, as an example, PA410/Pebax/CNT and PA410/PA6/Pebax/CNT NCs containing 0.6 wt% CNTs. Curves have been shifted in the vertical axis.

Figure 2 and Table 3 and Table 4 show the results obtained by DSC. On the one hand, Figure 2 shows the DSC curves of pure PA410, PA410/Pebax 70/30 unfilled blend and, as an example, PA410/Pebax/CNT and PA410/PA6/Pebax/CNT NCs with a 0.6 wt% CNT content. On the other hand, Table 3 and Table 4 show the main calorimetric parameters of the PA410/Pebax/CNT and the PA410/PA6/Pebax/CNT systems, respectively, i.e., the melting temperatures ($T_{m}$), enthalpies ($∆H_{m}$), and the crystalline levels ($\chi_{c}$) from the first heating scans and the crystallization temperatures ($T_{c}$) and enthalpies ($∆H_{c}$) from the cooling scans, respectively. According to the heating scan of the ternary system, the melting temperature of the PA410 barely changes after the addition of the CNTs with respect to the pure PA410; however, the normalized melting enthalpy increases slightly. These results agree with the results obtained for the corresponding binary systems, i.e., PA410/CNT [1] and PA410/Pebax (Table S2). This is because the increasing CNT content hardly affected the $T_{m}$, and a similar nucleation effect to that observed in the present system was observed as a result of adding Pebax to the pure PA410.

With respect to the cooling scan, the CNTs maintain in the ternary system the same clear nucleation effect upon the $T_{c}$ and the $∆H_{c}$ of the PA410 (increases of 9 °C and 20–40%, respectively) shown in the corresponding binary PA410/CNT system [1]. In the corresponding PA410/Pebax binary system, the elastomer particles showed a slight nucleation effect on the PA410 matrix (Table S2). The results show that the significant nucleation effect of the CNTs covers up that slight effect. In conclusion, the changes observed in crystallization parameters are mainly caused by the CNTs.

Regarding the PA410/PA6/Pebax/CNT quaternary system (Table 4), significant changes are observed in $T_{m}$, and very slight increases in $∆H_{m}$. The discussion of these results is more complex because combined effects may arise as a consequence of the joint presence of PA6, Pebax, and CNTs within the system. If a comparison is made with respect to the PA410/PA6/CNT ternary system [46] (i.e., the system without Pebax), the behavior is quite similar. This is because the miscible PA6 caused a significant decrease in the $T_{m}$ of the PA410 [46], while no effect was observed as a result of the addition of the CNTs [46]. However, neither the PA6 nor the CNTs significantly affected the $∆H_{m}$. Thus, the slight increase observed in the quaternary system can be attributed to the Pebax, which is consistent with the results obtained in the corresponding PA410/Pebax binary system (Table S2).

As far as crystallization parameters are concerned (i.e., $T_{c}$ and $∆H_{c}$), the behavior of the quaternary system is very similar to that of the PA410/PA6/CNT ternary system [46] (i.e., the system without Pebax), as both parameters show significant increases. Therefore, it is concluded that the nucleation effect of the CNTs over the crystallization of the PA410 is maintained, and that the presence of Pebax does not affect that behavior.

In conclusion, although the growing complexity of the studied systems makes the comparison with the preceding systems more difficult, the phase structure of the PA410/Pebax/CNT and the PA410/PA6/Pebax/CNT systems is the sum of a simple addition of the effects of each component, as no particular effect has been observed as a result of their joint presence.

### 3.2. Nanostructure and Morphology

**Figure 3:**
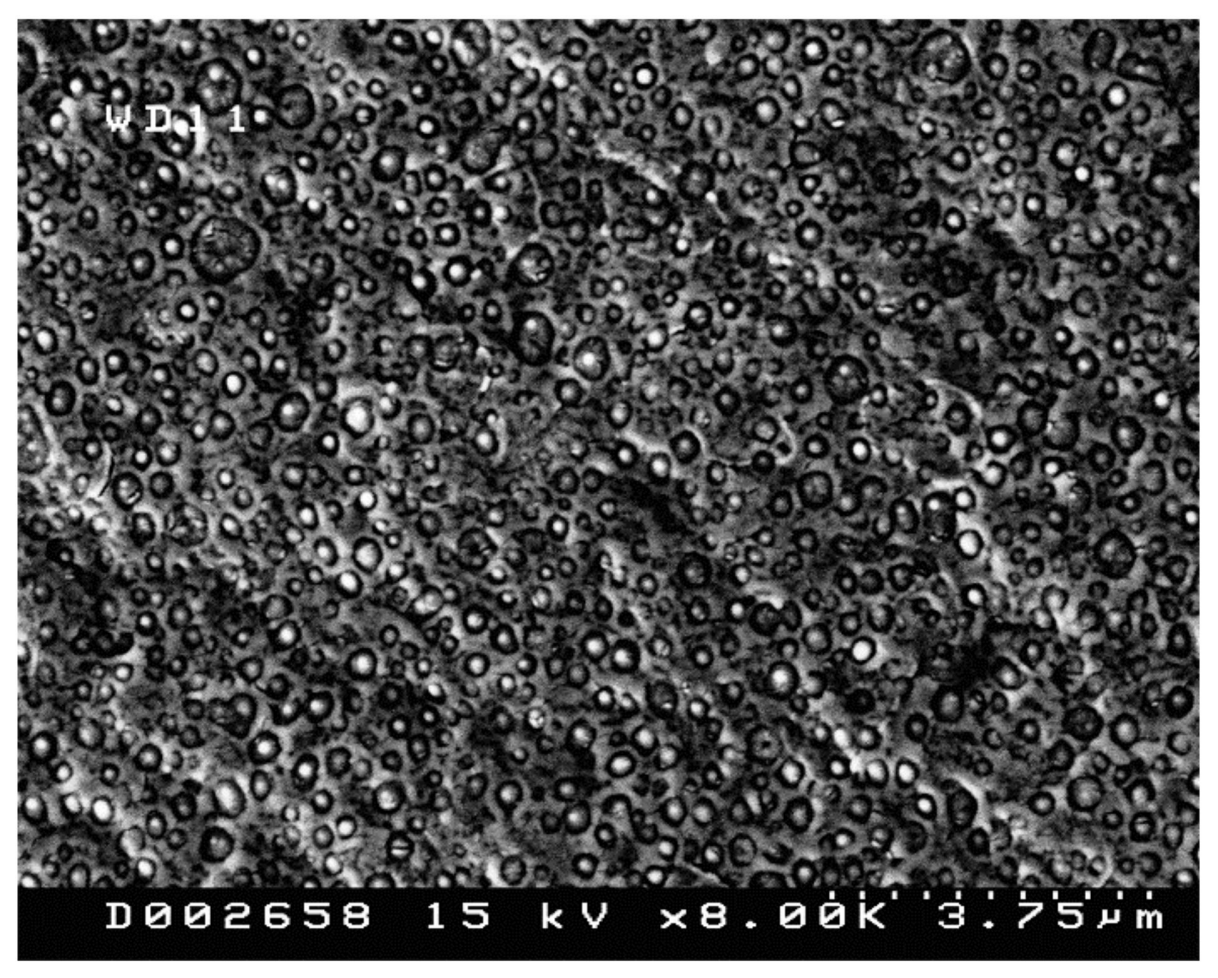
SEM micrograph of unfilled PA410/Pebax 70/30 blend.

In the preliminary studies, the matrix and dispersed phase morphology of the PA410/Pebax immiscible binary system has been described for compositions up to a Pebax content of 40%, and in particular, for the 70/30 PA410/Pebax composition (Figure 3 and Figure S2). In the case of the PA410/Pebax/CNT and PA410/PA6/Pebax/CNT systems, two main aspects must be discussed: (1) whether the presence of CNTs (in the ternary system) and the joint presence of PA6 and CNTs (in the quaternary system) affects the matrix and dispersed phase morphology of the PA410/Pebax 70/30 blend, and (2) the distribution of the CNTs between the two phases present in the systems under study (i.e., PA410 and Pebax in the case of the ternary system, and PA410/PA6 and Pebax in the quaternary system).

In order to facilitate the discussion of the results, the distribution of the CNTs between the phases is discussed first, since the presence of the CNTs in either of the phases or both phases, or in the interphase can be decisive in the final morphology of multiphasic systems [48,49,50].

**Figure 4:**
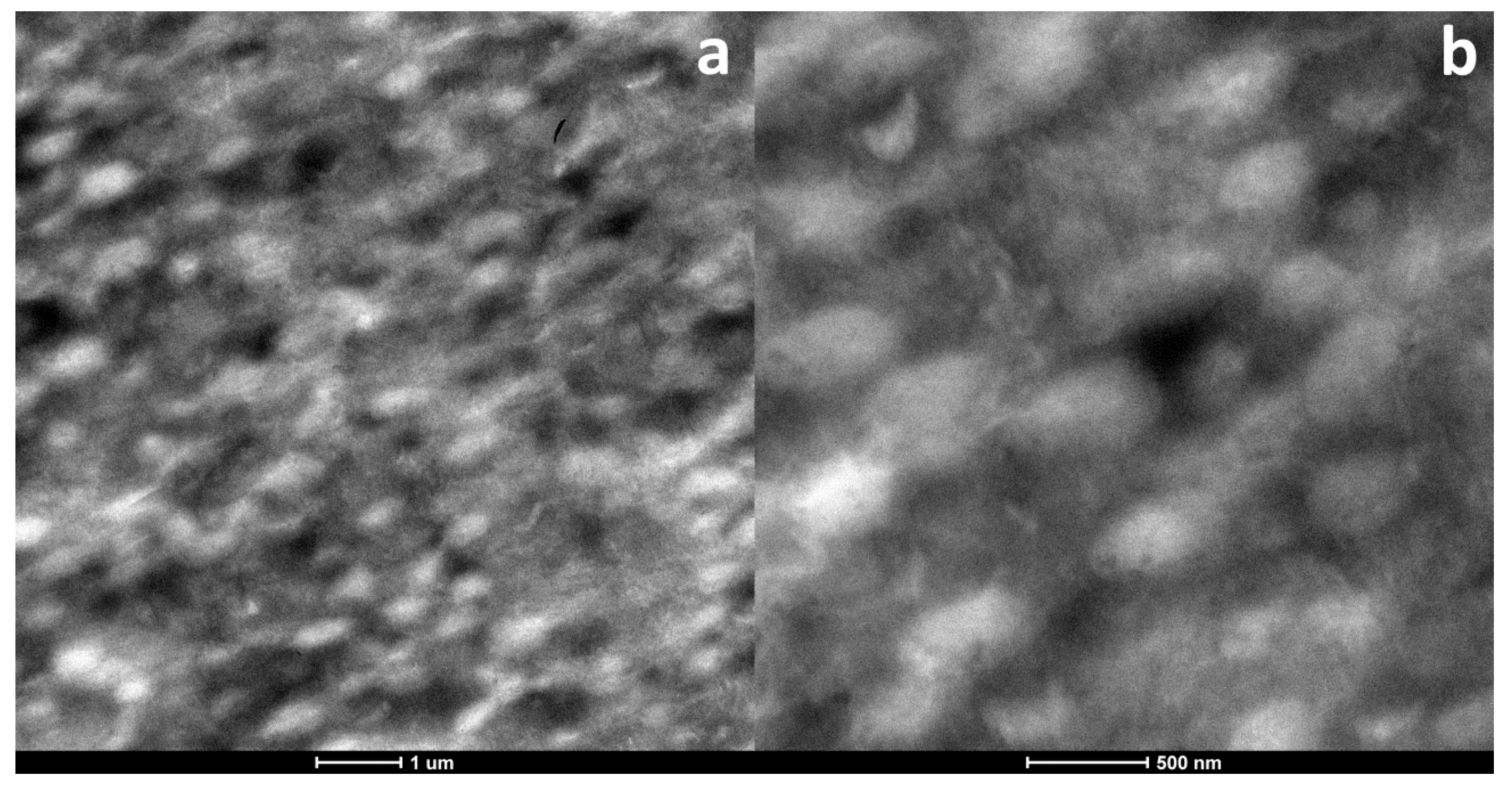
TEM micrographs of the PA410/Pebax 70/30 blend: (**a**) At ×5000 magnification; (**b**) at ×14,500 magnification.

**Figure 5:**
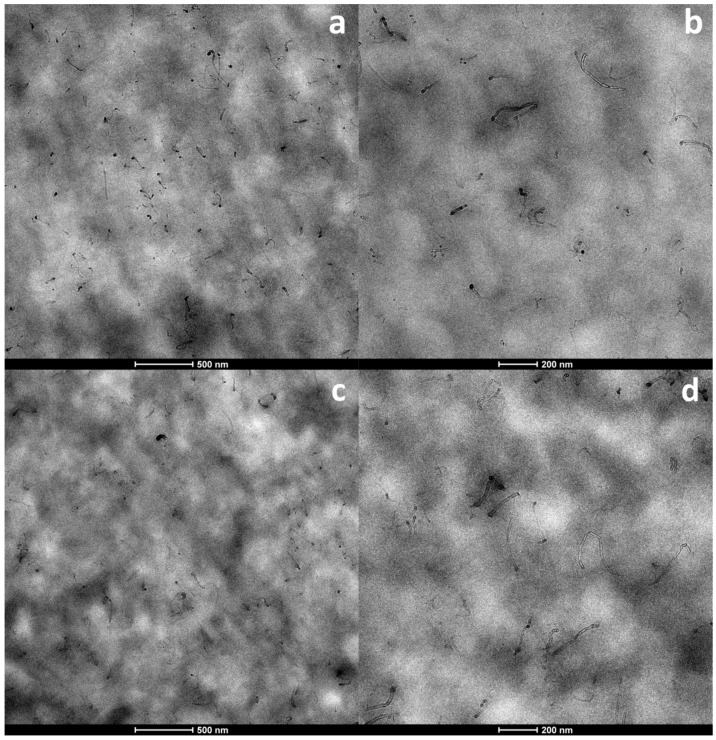
TEM micrographs of PA410/Pebax/CNT ternary (**a**,**b**) and PA410/PA6/Pebax/CNT quaternary (**c**,**d**) NCs with a CNT content of 0.4 wt%, at ×14,500 and ×25,000 magnifications.

Figure 4 and Figure 5 show, respectively, the TEM micrographs of unfilled PA410/Pebax 70/30 blend and of PA410/Pebax/CNT ternary (Figure 5a,b) and PA410/PA6/Pebax/CNT quaternary (Figure 5c,d) NCs with a CNT content of 0.4 wt%, at ×14,500 and ×25,000 magnifications. It should first be mentioned that the presence of CNTs reduces the contrast between the two polymeric components (polyamide-based matrix and Pebax) with respect to the unfilled polyamide/Pebax binary systems (see Figure 4), due to the high contrast between the CNT and any polymeric component. Although not as clear as in the unfilled binary system, the matrix and dispersed elastomeric phases can still be distinguished, the former being darker than the latter. When the CNT distribution is examined, they seem to be mainly located in the PA410 or PA410/PA6 continuous phase of the ternary and quaternary systems, respectively. Some of them can also be observed in the Pebax dispersed phase but in a clearly lower amount. This indicates that CNTs have a higher chemical affinity for the polyamide phase than for the Pebax phase. Indeed, it is widely known that the more polar the matrix, the higher the affinity of the CNTs for that matrix [51,52,53]. In fact, several studies have shown that CNTs in thermoplastic/thermoplastic/CNT systems tend to locate in the most polar phase [30,31,52,54], and in PA/elastomer/CNT NCs, in particular, they tend to locate in the PA phase [30,31]. Thus, the CNTs, even the ones that are not chemically modified [52], often show high affinity for the polyamide phase because the interfacial tension is usually lower between the PA and the CNTs [52]. If we consider the chemical structure of the polymeric components that are present in the systems under study, PA410, PA6, and Pebax include highly polar amide groups within their structure, which explains the affinity of CNTs for both the PA phase and the Pebax phase. However, the density of amide groups is not the same in all the components; PA6 has the highest amide group density, closely followed by PA410, and, lastly, Pebax, with the lowest amide group density, which is composed of polyether in 52–56% and where the amide groups are separated by 11 carbon atoms.

Hence, the affinity of the CNTs for the components of the system explains the presence of CNTs in both phases and the fact that their content is higher in the polyamide continuous phase (i.e., PA410 or PA410/PA6) than in the Pebax phase. In biphasic CNT-filled systems, the CNTs can be located either in the matrix [30,31,52,54], in the dispersed phase [55,56,57], or in the interphase [51,52,53,54,55,56,57,58], which is normally established by the affinity of the CNTs for each of the components, although the processing sequence can also have an influence [30,31,53,54]. Zhang et al. [52], for instance, studied PA6/PP/CNT NCs, and observed that the CNTs were located in the PA6 matrix. They measured the interfacial tension and observed that it was lower in the PA6/CNT interphase than in the PP/CNT interphase. Thus, CNTs had a higher affinity for the PA6 matrix than for the PP dispersed phase. They inferred from the results that this fact, as well as the processing sequence employed, resulted in the location of the CNTs in the PA6 matrix. Similar results have been obtained in several other PA6-based ternary NCs [30,31,54]. Cheng et al. [58] studied PA6/liquid crystalline polymer (LCP)/CNT NCs, and observed that CNTs were located in the PA6 matrix, and also in the interphase, owing to the strong interfacial adhesion between the CNTs and the two polymers. Hence, the CNTs acted as compatibilizers in the immiscible PA6/LCP blends, which improved the general mechanical performance of the NCs. Wu et al. [51] observed similar results in their PCL/PLA/CNT NCs. Furthermore, Urquijo et al. [55] studied PLA/PBAT/CNT NCs, and observed that CNTs were located exclusively in the PBAT dispersed phase. They measured the interfacial tension and observed that it was lower in the PBAT/CNT interphase than in the PLA/CNT interphase. Similar results have been obtained in many other studies with dispersed phases such as PBAT [56,57] and PCL [59]. Sometimes the CNTs can be located both in the matrix and in the dispersed phase. For example, Pötschke et al. [60] prepared PC/PE/CNT NCs by adding PE to a PC/CNT masterbatch. They observed that in the ternary NCs part of the CNTs were located in the PE dispersed phase. Indeed, CNTs bridged the PC matrix and the PE dispersed phase, since the length of the CNTs was higher than the phase sizes in the blends.

**Figure 6:**
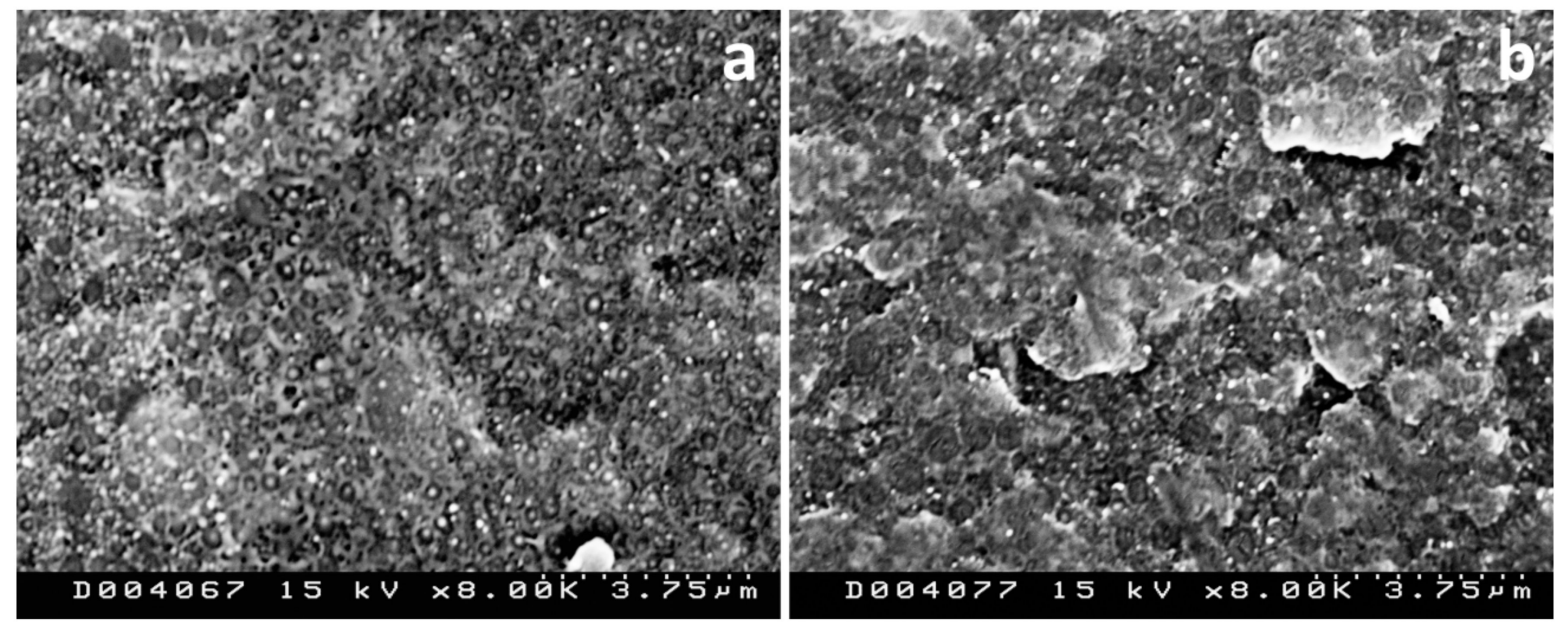
SEM micrographs of PA410/Pebax/CNT NCs: (**a**) 0.6% CNT; (**b**) 1% CNT.

**Figure 7:**
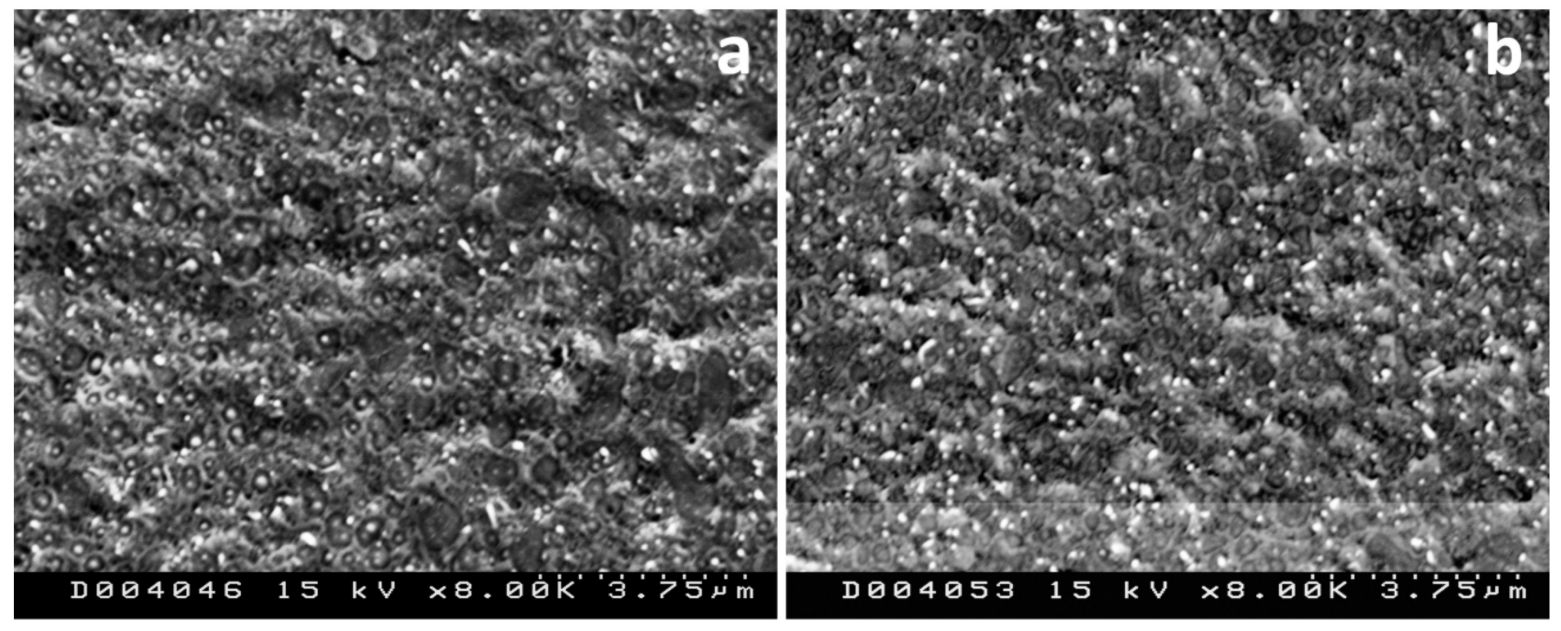
SEM micrographs of PA410/PA6/Pebax/CNT NCs: (**a**) 0.6% CNT; (**b**) 1% CNT.

With respect to the matrix-dispersed phase morphology of the systems under study, Figure 6 and Figure 7 show, respectively, the SEM micrographs of the PA410/Pebax/CNT and the PA410/PA6/Pebax/CNT NCs corresponding to the compositions with 0.6% and 1% of CNTs. As can be seen, the sea-island morphology described for the PA410/Pebax2 binary system is maintained in both cases despite the presence of CNTs, and they seem to have no influence either on the shape or on the size of the dispersed phase (see Figure 3).

In conclusion, in both systems under study (PA410/Pebax/CNT and PA410/PA6/Pebax/CNT), the CNTs are located both in the matrix (mainly) and in the dispersed phase. This would explain the lack of effect of the CNTs on the size of the dispersed phase, in view of the fact that the usual effects caused by the location of the CNTs in the continuous phase (i.e., decrease of the particle size) would offset the usual effects caused by the location of the CNTs in the dispersed phase (i.e., increase of the particle size). Finally, it must be mentioned that the geometry of the dispersed phase remains unchanged as well, even if the presence of CNTs in the dispersed phase usually causes changes in its geometry [50,61]. The stability of the dispersed phase geometry is probably due to (a) the presence of CNTs not only in the dispersed phase, but also in the matrix and (b) the lower concentration of CNTs in the dispersed phase. Pötschke et al. [60], for instance, also observed that in their PC/PE/CNT NCs, where CNTs were located both in the PC matrix and in the PE dispersed phase, the CNT content did not affect the morphology of the NCs.

### 3.3. Electrical Properties

**Figure 8:**
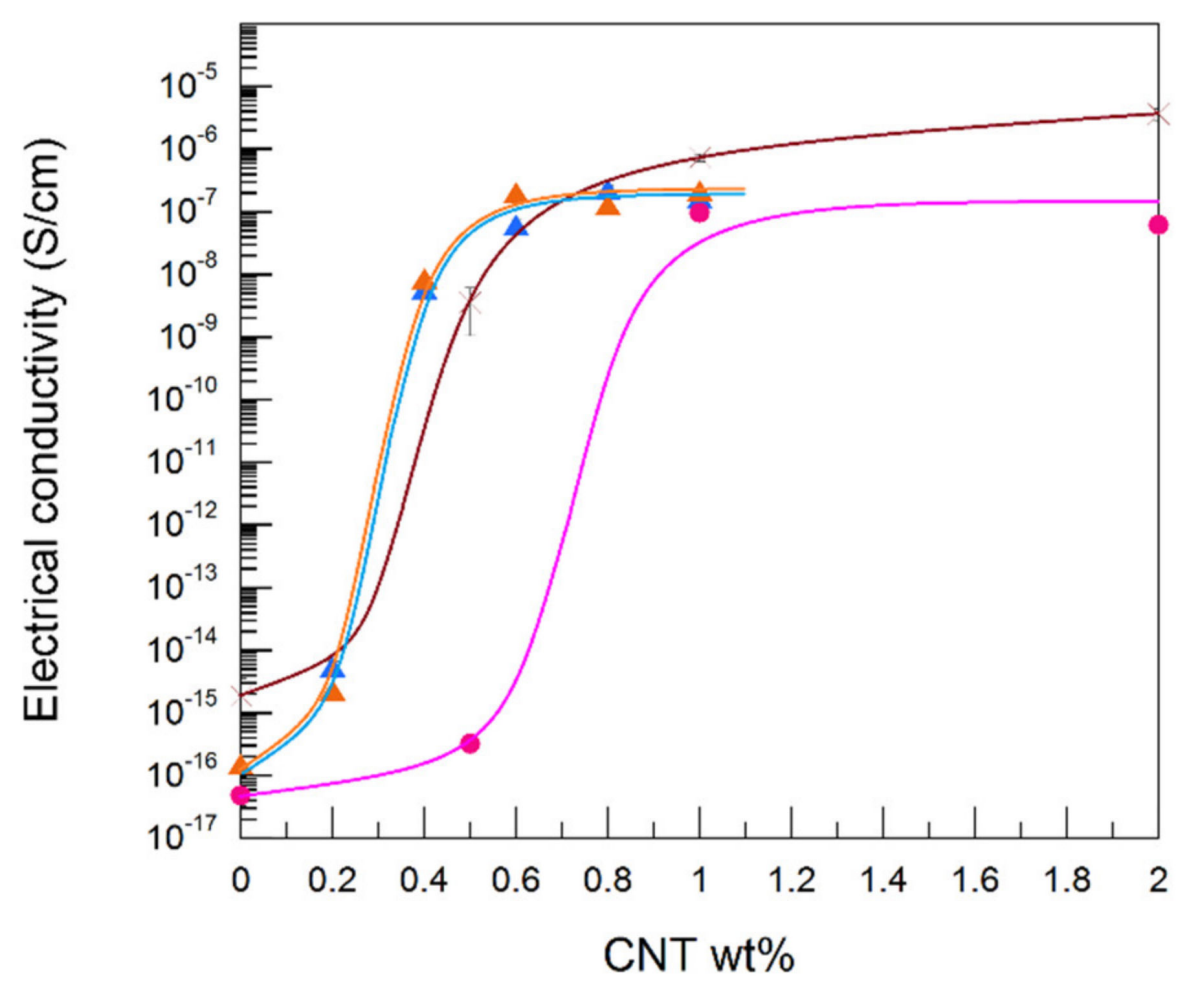
Electrical conductivity of PA410/Pebax/CNT (▲) and PA410/PA6/Pebax/CNT (▲) NCs vs. the CNT content. PA410/CNT (x) and PA410/PA6/CNT (●) systems have been represented as a reference [1].

Figure 8 shows the electrical conductivity values of the PA410/Pebax/CNT and the PA410/PA6/Pebax/CNT systems vs. the CNT content. In order to facilitate the comparison between the systems under study (PA410/Pebax/CNT and PA410/PA6/Pebax/CNT) and the preceding corresponding systems without Pebax already studied in a previous work [1], the latter have also been included in the Figure. The electrical percolation concentration (p_c_) values of the four systems represented in Figure 8 are summarized in Table 5. As can be seen in both Figure 8 and Table 5, the p_c_ values of the two systems containing Pebax are very similar, and lower than those of the systems without Pebax. As discussed in our previous work [1], the dispersion level of the CNTs was very similar in the two systems without Pebax independent of the PA410 or PA410/PA6 nature of the matrix, as well as the aspect ratio of the CNTs. As a result, p_c_ values were similar in both systems. Therefore, the lower p_c_ values observed in the systems containing Pebax must, necessarily, be related to the presence of this component. Given the lower CNT content in the Pebax dispersed phase than in the PA continuous phase owing to the higher affinity of the CNTs for the polyamide phase (as stated in the previous section), a partial exclusion effect of the Pebax dispersed phase over the CNTs is proposed. The effective CNT concentration is higher in the continuous PA phase, and therefore the electrical percolation occurs at lower overall CNT concentrations.

The volume-exclusion effect has been observed in many other thermoplastic/thermoplastic/CNT systems. For example, Gonzalez et al. [53] studied PC/mSEBS/CNT NCs, where the CNTs were located exclusively in the PC phase, and observed that the p_c_ decreased from 0.5% in the PC/CNT NCs to 0.45% after the addition of 10% of mSEBS. Bin et al. [62] studied UHMWPE/LMWPE/CB ternary NCs (i.e., ultra-high molecular weight polyethylene/low molecular weight polyethylene/carbon black NCs). They observed that CB particles were located exclusively in the LMWPE. As a result, the percolation threshold was much lower than in the UHMWPE/CNT or in the LMWPE/CNT binary NCs. Similar results have been obtained in systems in which the so-called double percolation has been observed [63,64]. The double percolation implies that one of the phases is percolated within the other phase, and that the CNTs are percolated within one of the phases. This implies that the CNT-free phase exerts a volume-exclusion effect over the CNT-filled phase, and therefore the effect is analogous. With respect to PA/elastomer/CNT systems, Gonzalez et al. [30] studied PA6/mSEBS/CNT NCs, and observed that the p_c_ of the PA6/CNT binary NCs decreased from 4–5% to 3.5% after the addition of the elastomer, because, in the ternary NCs, the CNTs were located exclusively in the PA6 phase, and the percolation threshold decreased due to the volume-exclusion effect exerted by the elastomer. Dasari et al. [31] studied PA6/POE-g-MA/CNT NCs, where the CNTs were located in the PA6 phase as well. The percolation threshold was around 2.5–5% CNT, and in this case, the volume-exclusion effect was noticed in the electrical conductivity values, because the conductivity values of the ternary NCs were significantly higher than those of the corresponding binary PA6/CNT NCs. According to the authors, the volume of PA6 available for CNTs to occupy decreased due to the addition of the elastomer, and hence the CNT concentration was higher in the continuous PA phase. Because of the volume-exclusion effect, the electrical conductivity values were higher in the ternary NCs than in their corresponding binary NCs. Similar results have been observed in several other studies [65,66].

Regardless of the effect on the mechanical properties, which are discussed below, the addition of Pebax to the PA410/CNT and the PA410/PA6/CNT systems has a positive effect on the electrical performance of the corresponding systems, as the percolation threshold is achieved at lower CNT contents due to the partial exclusion effect of the Pebax phase.

### 3.4. Mechanical Properties

**Figure 9:**
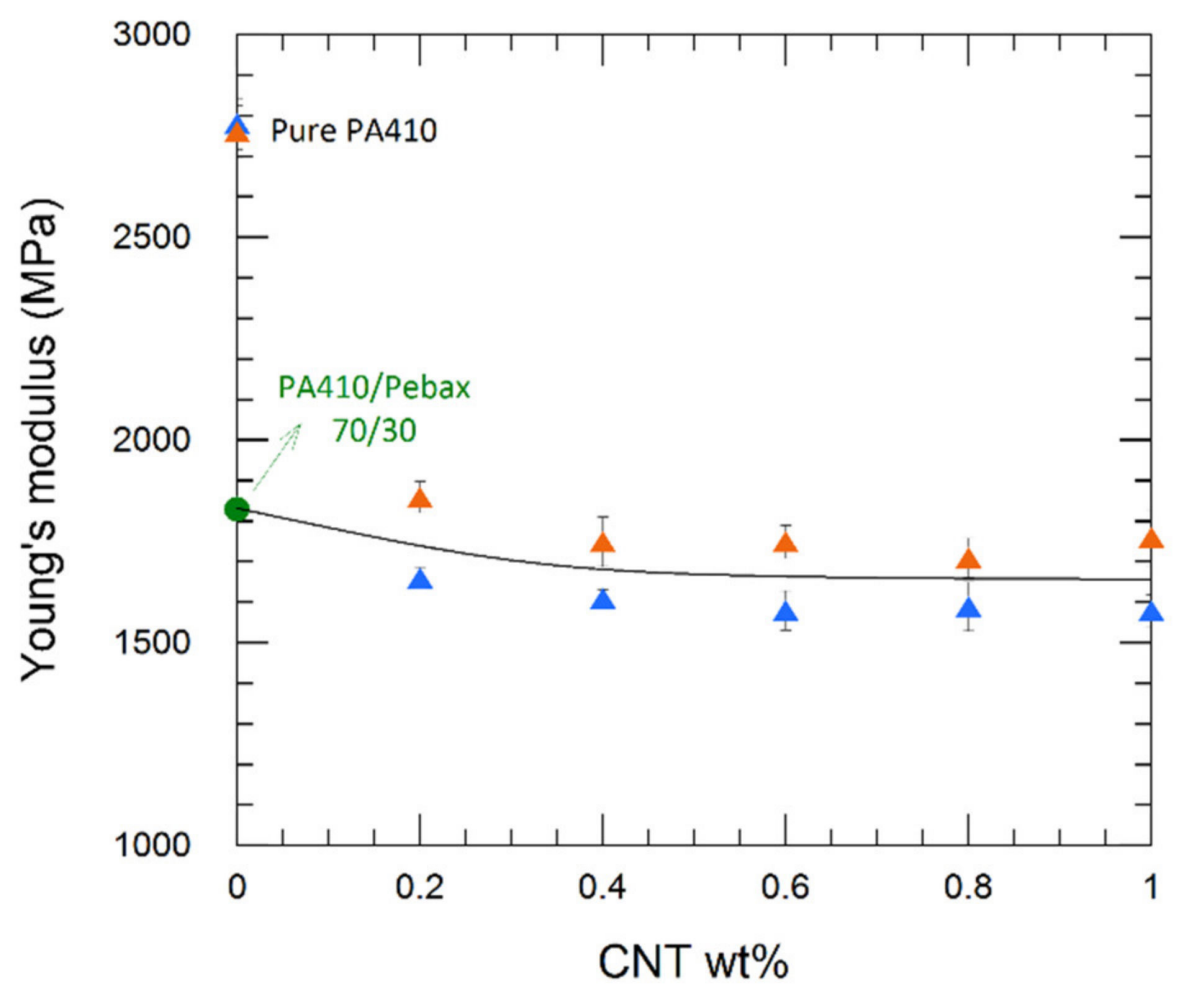
Young’s modulus of PA410/Pebax/CNT (▲) and PA410/PA6/Pebax/CNT (▲) NCs vs. the CNT content. Pure PA410 and the PA410/Pebax2 70/30 blend have been represented as a reference.

**Figure 10:**
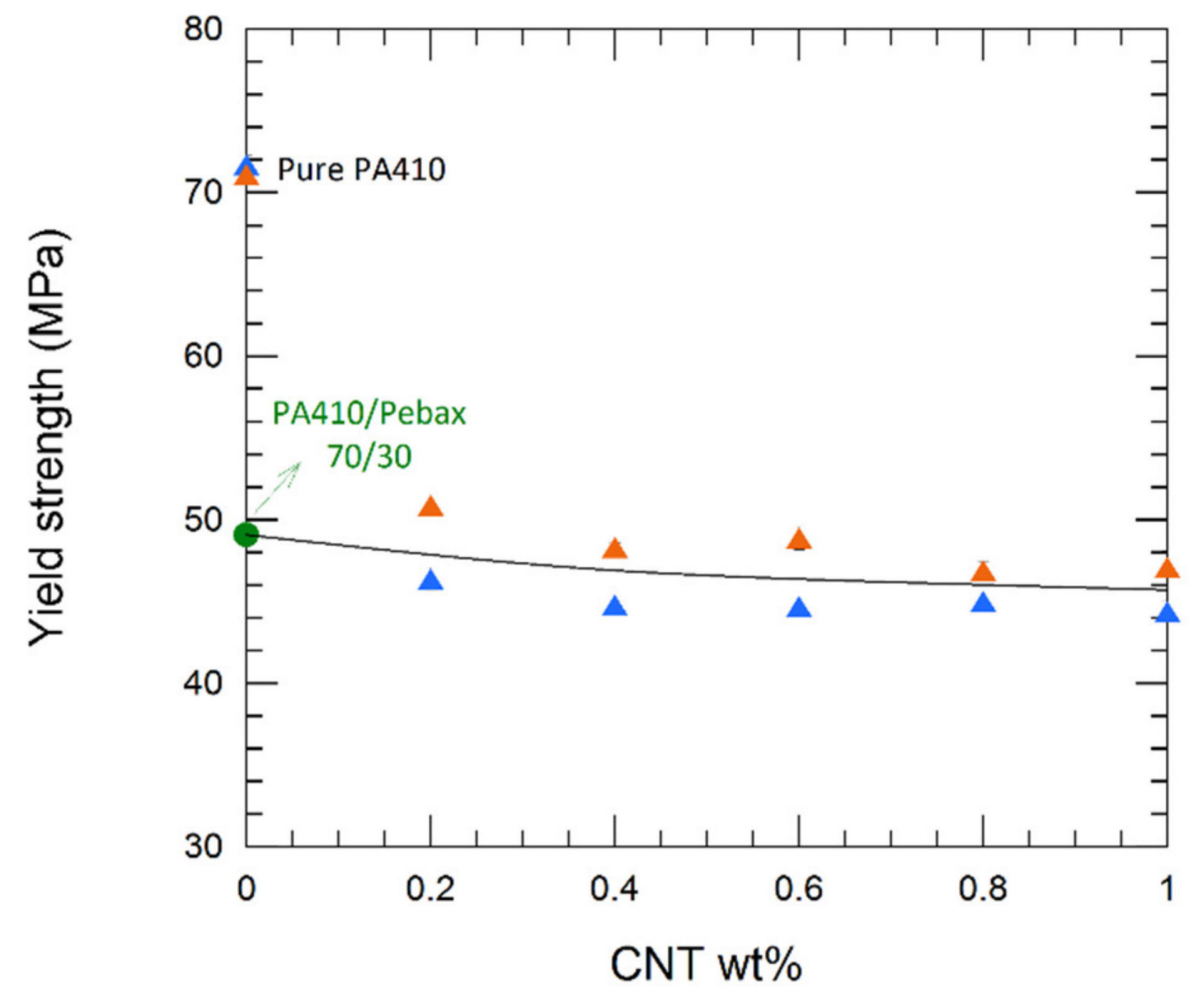
Yield strength of PA410/Pebax/CNT (▲) and PA410/PA6/Pebax/CNT (▲) NCs vs. the CNT content. Pure PA410 and the PA410/Pebax2 70/30 blend have been represented as a reference.

Figure 9 and Figure 10 show the values for the Young’s modulus and yield strength properties, respectively, of the ternary PA410/Pebax/CNT NCs and the quaternary PA410/PA6/Pebax/CNT NCs vs. the CNT content. The values corresponding to the pure PA410 and the unfilled PA410/Pebax 70/30 blend are also represented as a reference. As has been previously demonstrated [30,53], these two properties showed a similar behavior, that is, a significant decrease with respect to the pure PA410, which was approximately proportional to the Pebax content and ascribed to its elastomeric nature. In relation to the effect of the addition of the CNTs, the values for both Young’s modulus and yield strength remain almost constant in the two systems regardless of the CNT content, and the values are slightly lower with respect to the reference PA410/Pebax 70/30 blend. This behavior is unusual; indeed, when CNTs are added to a polymeric matrix, low deformation mechanical properties usually increase [30,53]. However, the CNT contents of the systems under study must be considered very low (below 1 wt%). In fact, in polymer/CNT NCs with such low CNT content, the lack of improvement in mechanical properties is not unusual [67,68,69], especially considering that the strengthening efficiency of the CNTs is significantly lower in stiff polymers [70], and that depending on the dispersion level of the nanofiller, the modulus could even decrease [70]. Furthermore, in the corresponding systems without Pebax studied in our previous work (i.e., PA410/CNT and PA410/PA6/CNT) [1], an improvement in Young’s modulus was observed with CNT contents above 2–3 wt%, while lower CNT contents led to slight decreases in modulus, as observed in the systems in the present work.

**Figure 11:**
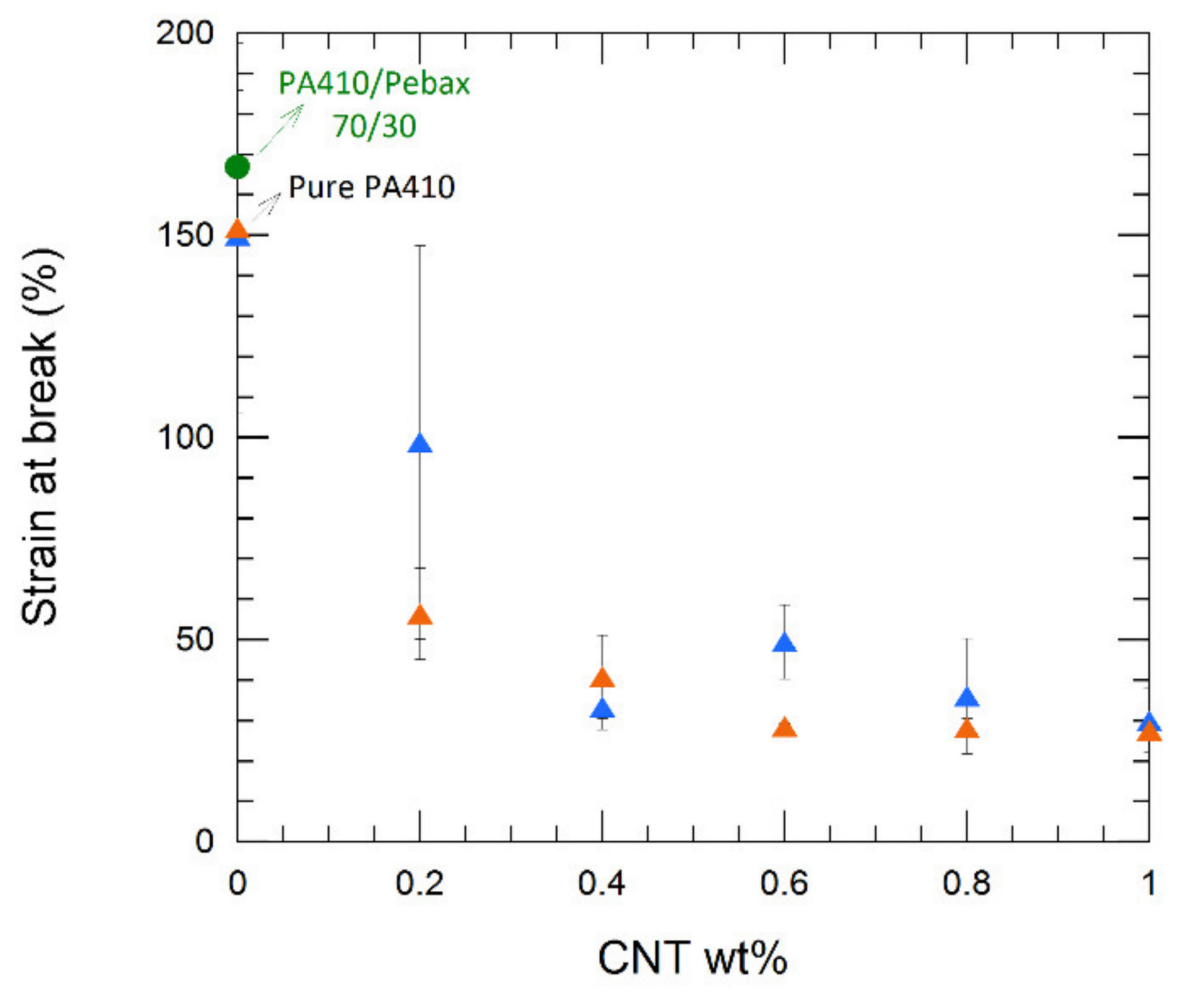
Strain at break of PA410/Pebax/CNT (▲) and PA410/PA6/Pebax/CNT (▲) NCs vs. the CNT content. Pure PA410 and the PA410/Pebax2 70/30 blend have been represented as a reference.

Regarding the deformability of the systems under study, Figure 11 represents the strain at break values vs. the CNT content. Again, the values corresponding to the pure PA410 and the PA410/Pebax 70/30 blend are represented as a reference. As expected, the deformability decreases sharply after CNT addition, even at CNT contents as low as 0.4%. This happens because CNTs promote failure of the continuous phase, i.e., PA410 or PA410/PA6, acting as predecessors of fracture in this type of notch sensitive [36,71,72] and pseudo-ductile matrixes. The performance is very similar in the ternary PA410/Pebax/CNT and in the quaternary PA410/PA6/Pebax/CNT systems; the presence of PA6 has no effect on either the deformability or on the notch sensitivity of the majority PA410.

Nevertheless, the effect of Pebax on the performance of these systems is noteworthy since even if the deformability decreases as a result of the addition of CNTs, all the compositions remain ductile, with strain at break values over 30%. It is indeed a remarkable improvement with respect to the systems without Pebax, which were brittle in the whole CNT-composition range studied [1] and showed strain at break values below 10%.

**Figure 12:**
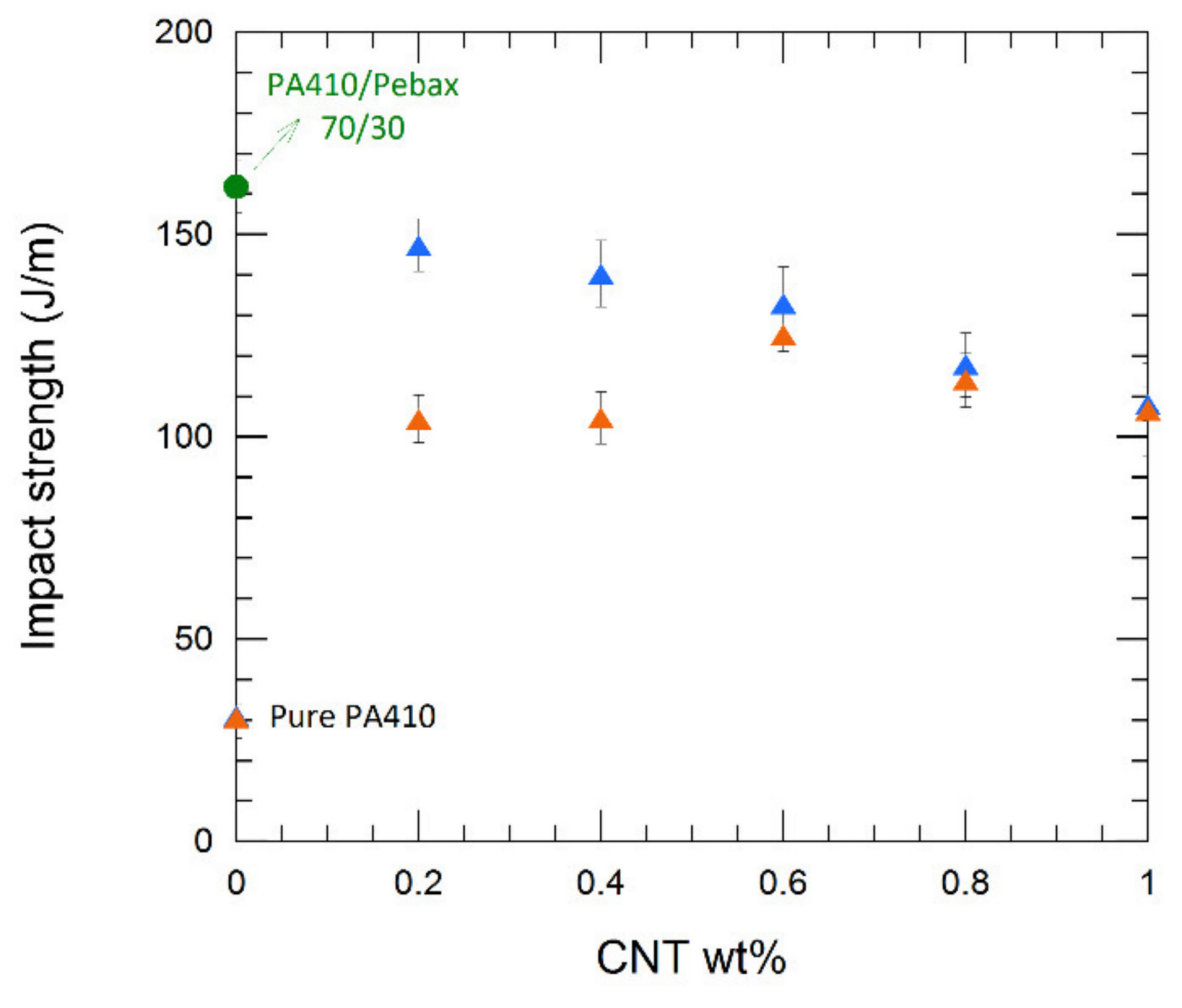
Impact strength of PA410/Pebax/CNT (▲) and PA410/PA6/Pebax/CNT (▲) NCs vs. the CNT content. Pure PA410 and the PA410/Pebax2 70/30 blend have been represented as a reference.

Finally, Figure 12 shows the notched impact strength values of the PA410/Pebax/CNT and the PA410/PA6/Pebax/CNT systems vs. the CNT content. The values corresponding to the pure PA410 and the PA410/Pebax 70/30 blend are represented as a reference. As expected, and as with the strain at break, CNTs have a negative effect on the capability of these systems to absorb energy during deformation or fracture processes in comparison to the unfilled PA410/Pebax 70/30 blend. Nonetheless, in the case of impact strength, the comparison with the pure PA410 is very favorable. In fact, the PA410 shows a very low impact strength resulting from its high notch sensitivity, but the Pebax containing NCs show values that are from three to five-fold higher than that of the pure PA410. Still, and as mentioned in the case of the unfilled PA410/Pebax system, the results are not even close to the super-toughness observed in other PA/elastomer/CNT systems [30,31].

No clear conclusions can be drawn from the comparison between the ternary and the quaternary systems. This is because, at low CNT contents, the values of the PA410/PA6/Pebax/CNT system are slightly higher than those of the PA410/Pebax/CNT system, but, at CNT contents above 0.6%, the values of both systems are very similar, and the differences are close to the experimental error of the measurements.

## 4. Conclusions

The combined addition of 30% Pebax and CNTs (either in the form of pristine nanotubes or PA6-based masterbatch) to the PA410 results in NCs with improved electrical properties, i.e., lower percolation thresholds, which is attributed to the lower CNT concentration needed to achieve electrical percolation due to the partial volume exclusion effect of the dispersed Pebax over the CNTs. Moreover, the mechanical properties attained are of considerable interest, since all the materials remain ductile, and the impact strength increases from three to five-fold with respect to the pure PA410, despite the usual decrease observed in low deformation mechanical properties (i.e., stiffness and yield strength). It is noteworthy, for instance, that, after the addition of only 0.6% CNTs, the system turns semiconductive, with an impact strength loss of only 20% with respect to the corresponding unfilled PA410/Pebax 70/30 blend but a 300% increase with respect to pure PA410. It is also remarkable that the addition method used to incorporate the CNTs to the system does not affect the final properties of the NCs, pointing to the beneficial use of the masterbatch regarding its manipulation.

## Figures and Tables

**Table 1:** PA410, PA6, Pebax, and CNT wt% contents in the compositions studied.

	Nomenclature	PA410 (wt%)	PA6 (wt%)	Pebax (wt%)	Nanocyl NC7000^TM^ (wt%)
PA410/Pebax/CNT ternary NCs	100/0/0	100	-	0	0
	69.8/30/0.2	69.8	-	30	0.2
	69.6/30/0.4	69.6	-	30	0.4
	69.4/30/0.6	69.4	-	30	0.6
	69.2/30/0.8	69.2	-	30	0.8
	69/30/1	69	-	30	1
PA410/PA6/Pebax/CNT quaternary NCs	100/0/0/0	100	0	0	0
	68.7/1.1/30/0.2	68.7	1.1	30	0.2
	67.3/2.3/30/0.4	67.3	2.3	30	0.4
	66/3.4/30/0.6	66	3.4	30	0.6
	64.6/4.6/30/0.8	64.6	4.6	30	0.8
	63.3/5.7/30/1	63.3	5.7	30	1

**Table 2:** The α and β transition values of PA410/Pebax/CNT and PA410/PA6/Pebax/CNT NCs obtained from DMTA curves.

PA410/Pebax/CNT Composition	α (°C)	β (°C)	PA410/PA6/Pebax/CNT Composition	α (°C)	β (°C)
100/0/0	76.8 ± 0.6	−52.7 ± 2.5	100/0/0/0	74.8 ± 0.2	−54.3 ± 0.2
69.8/30/0.2	75.1 ± 0.7	−51.9 ± 0.0	68.7/1.1/30/0.2	74.1 ± 0.6	−51.4 ± 0.1
69.6/30/0.4	74.8 ± 0.5	−52.3 ± 0.3	67.3/2.3/30/0.4	74.4 ± 0.4	−51.8 ± 0.7
69.4/30/0.6	74.9 ± 0.2	−52.3 ± 1.5	66/3.4/30/0.6	72.3 ± 0.1	−53.2 ± 0.0
69.2/30/0.8	74.5 ± 0.2	−51.3 ± 0.5	64.6/4.6/30/0.8	72.9 ± 0.0	−50.7 ± 0.1
69/30/1	75.2 ± 0.4	−50.6 ± 0.3	63.3/5.7/30/1	72.0 ± 0.4	−54.0 ± 1.4

**Table 3:** Calorimetric parameters of the PA410/Pebax/CNT NCs. ^1^First heating scan. Values in brackets are normalized with respect to the weight fraction of PA410.

PA410/Pebax/CNT Composition	$T_{m}^{1}\;({}^{\circ}C)$	$∆H_{m}^{1}\;(J/g)$	$\chi_{c}\;(\%)$	$T_{c}\;({}^{\circ}C)$	$∆H_{c}\;(J/g)$
100/0/0	254.4	62.0	23	224.3	−37.9
69.8/30/0.2	255.1	49.1 (70.4)	26	230.9	−30.5 (−43.7)
69.6/30/0.4	254.1	45.3 (65.0)	24	232.9	−31.3 (−44.9)
69.4/30/0.6	254.7	47.3 (68.1)	25	233.3	−35.2 (−50.7)
69.2/30/0.8	253.1	46.3 (66.8)	25	233.3	−35.3 (−50.9)
69/30/1	254.4	48.2 (69.9)	26	233.9	−36.2 (−52.4)

**Table 4:** Calorimetric parameters of the PA410/PA6/Pebax/CNT NCs. ^1^First heating scan. Values in brackets are normalized with respect to the weight fraction of the polyamide phase.

PA410/PA6/Pebax/CNT Composition	$T_{m}^{1}\;({}^{\circ}C)$	$∆H_{m}^{1}\;(J/g)$	$\chi_{c}\;(\%)\;(*)$	$T_{c}\;({}^{\circ}C)$	$∆H_{c}\;(J/g)$
100/0/0/0	258.1	62.1	23	224.6	−36.7
(*) 68.7/1.1/30/0.2	254.4	45.6 (65.4)	-	232.6	−30.7 (−44.0)
67.3/2.3/30/0.4	252.7	43.4 (62.4)	-	233.6	−33.7 (−48.4)
66/3.4/30/0.6	253.4	43.2 (62.2)	-	233.9	−33.8 (−48.6)
64.6/4.6/30/0.8	252.1	43.5 (62.8)	-	233.6	−35.3 (−51.0)
63.3/5.7/30/1	254.4	44.5 (64.5)	-	233.9	−33.6 (−48.6)

(*) Crystallinity values are not reported because the matrix of the nanocomposites is composed of both PA410 and PA6.

**Table 5:** Electrical percolation concentration values of PA410/Pebax/CNT, PA410/PA6/Pebax/CNT, PA410/CNT, and PA410/PA6/CNT systems.

**System**	**p_c_ (wt%)**
PA410/Pebax/CNT	0.40
PA410/PA6/Pebax/CNT	0.38
PA410/CNT	0.50
PA410/PA6/CNT	0.65

## Supplementary Materials

The following are available online at https://www.mdpi.com/article/10.3390/polym13193420/s1, Table S1: Mechanical properties of PA410/Pebax1, PA410/Pebax2, and PA410/Pebax3 blends, Table S2: Calorimetric parameters of PA410, Pebax2, and PA410/Pebax2 blends obtained from the DSC curves, Table S3: Mechanical properties of PA410/Pebax2 blends, Figure S1: DMTA curves of pure PA410 and PA410/Pebax2 blends, Figure S2: SEM micrographs of cryogenically fractured surfaces of the PA410/Pebax2 blends: (a) 95/5; (b) 90/10; (c) 85/15; (d) 80/20; (e) 75/25; (f) 70/30; (g) 65/35; (h) 60/40.
