# Supplementary material for: Attaining Toughness and Reduced Electrical Percolation Thresholds in Bio-Based PA410 by Combined Addition of Bio-Based Thermoplastic Elastomers and CNTs
Source: Polymers (Basel). 2021 Oct 5;13(19):3420. doi: 10.3390/polym13193420 (PMC8512475; doi:10.3390/polym13193420)
Supplement: Supplementary file 1 [file polymers-13-03420-s001.zip › polymers-1404278-supplementary.pdf]

Supplementary information

Table S1. Mechanical properties of PA410/Pebax1, PA410/Pebax2 and PA410/Pebax3 blends.

	Composition	Young's modulus (MPa)	Yield strength (MPa)	Strain at break (%)	Impact strength (J/m)
PA410	100/0	2780±20	73.4±0.3	133±50	31±2
PA410/Pebax1	90/10	2350±20	63.2±0.5	36±36	62±2
	80/20	2050±30	53.4±1.1	110±61	113±2
	70/30	1840±20	46.0±0.9	121±17	162±5
PA410/Pebax2	90/10	2300±30	61.9±0.5	76±43	60±4
	80/20	2090±20	56.2±0.3	133±47	130±6
	70/30	1830±30	49.8±0.8	161±12	162±6
PA410/Pebax3	90/10	2430±70	63.0±0.9	122±64	39±3
	80/20	2120±90	61.8±0.9	103±53	60±1

Table S2. Calorimetric parameters of PA410, Pebax2 and PA410/Pebax2 blends obtained from the DSC curves. T_m^1 and ΔH_m^1, melting temperature and enthalpy, respectively, from the first heating scan. χ_c, crystallinity degree considering ΔH_f^{∞}=269 J/g[42]. T_c and ΔH_c, crystallization temperature and enthalpy, respectively, from the cooling scan. Values in brackets are normalized with respect to the weight fraction of PA410 in the blends.

PA410/Pebax2 composition	T_m^1(°C)	ΔH_m^1(J/g)	χ_c(%)	T_c(°C)	ΔH_c (J/g)
100/0	256.4	55	20	225.3	-38
95/5	252.4	59(62)	23	224.6	-39(41)
90/10	254.1	56(62)	23	223.6	-38(42)
85/15	253.7	49(58)	22	223.9	-35(41)
80/20	254.4	45(56)	21	223.6	-33(42)
75/25	252.4	46(61)	23	223.3	-32(43)
70/30	253.7	44(62)	23	222.9	-29(41)
65/35	254.4	37(58)	21	222.9	-24(37)
60/40	253.7	38(64)	24	223.3	-24(41)
0/100	157.1	24	---	104.6	-20

Table S3. Mechanical properties of PA410/Pebax2 blends.

PA410/Pebax2 composition	Young's modulus (MPa)	Yield stress (MPa)	Strain at break (%)	Impact strength (J/m)
100/0	2780±20	73.4±0.3	133±50	31±2
95/5	2460±40	66.5±0.9	33±8	43±1
90/10	2300±30	61.9±0.5	76±43	60±4
85/15	2230±20	59.1±0.9	124±33	100±5
80/20	2090±20	56.2±0.3	133±47	130±6
75/25	2000±20	53.1±0.5	137±56	143±9
70/30	1830±30	49.8±0.8	161±12	162±6
65/35	1680±30	46.8±0.5	62±13	122±10
60/40	1570±20	42.6±0.3	162±15	118±12
0/100	---	24.9±0.5	512±15	N/B

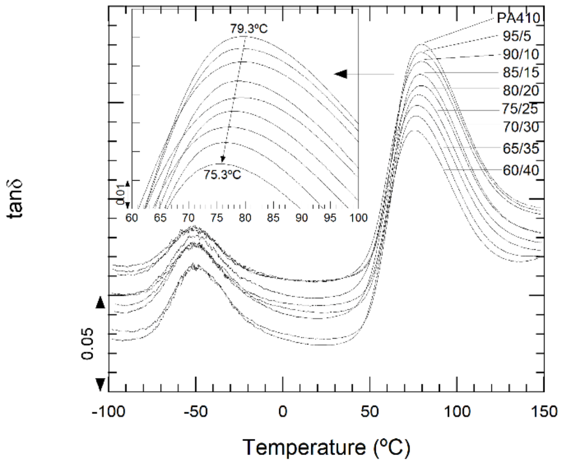

Figure S1. DMTA curves of pure PA410 and PA410/Pebax2 blends. Curves have been shifted in the vertical axis.

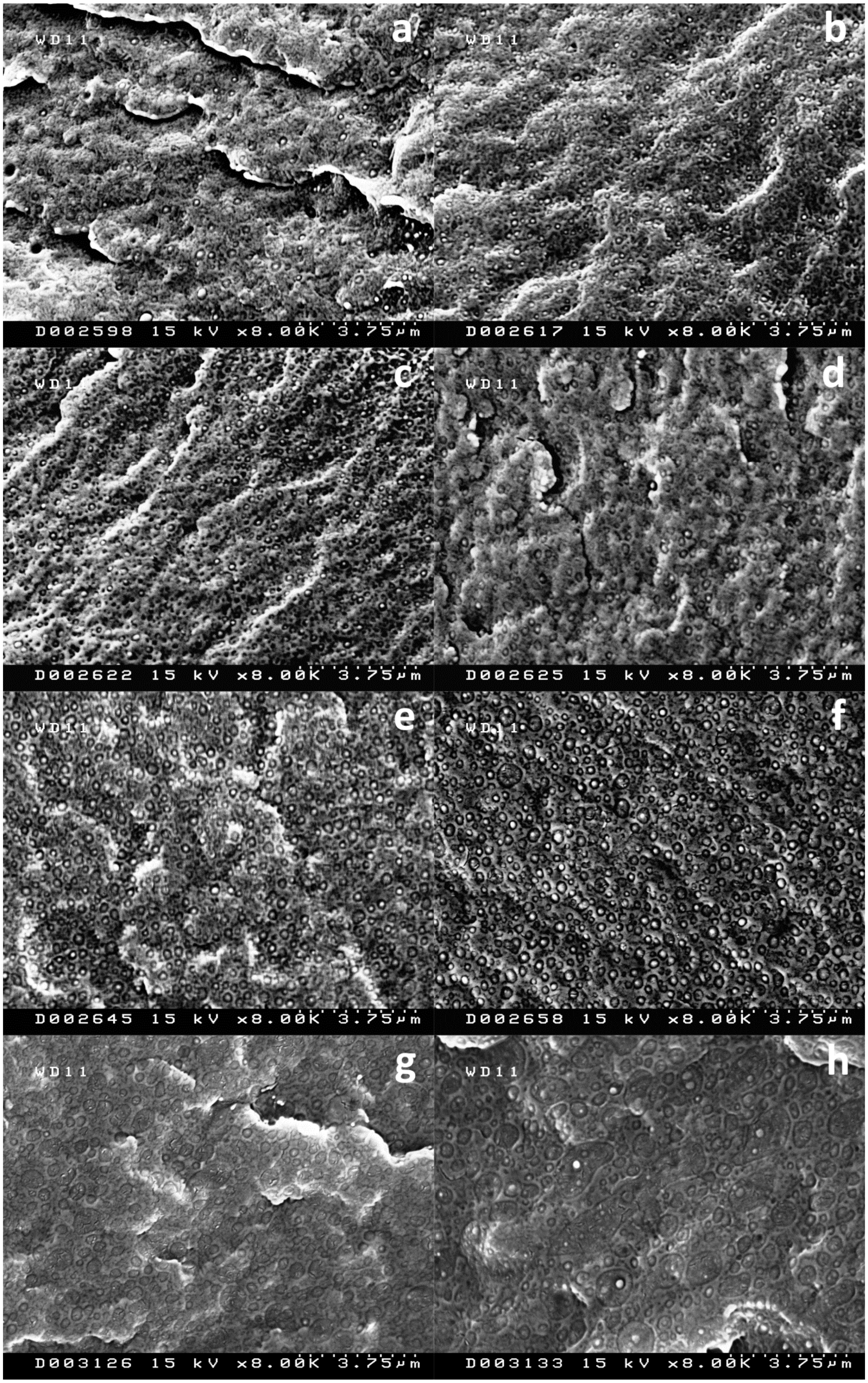

Figure S2. SEM micrographs of cryogenically fractured surfaces of the PA410/Pebax2 blends: (a) 95/5, (b) 90/10, (c) 85/15, (d) 80/20, (e) 75/25, (f) 70/30, (g) 65/35, (h) 60/40.

45. Moran, C. S.; Barthelon, A.; Pearsall, A.; Mittal, V.; Dorgan, J. R. Biorenewable blends of polyamide-4,10 and polyamide-6,10. J. Appl. Polym. Sci. 2016, 133, 43626. DOI: https://doi.org/10.1002/app.43626
